# Perturbation training to promote safe independent mobility post-stroke: study protocol for a randomized controlled trial

**DOI:** 10.1186/s12883-015-0347-8

**Published:** 2015-06-06

**Authors:** Avril Mansfield, Anthony Aqui, Andrew Centen, Cynthia J. Danells, Vincent G. DePaul, Svetlana Knorr, Alison Schinkel-Ivy, Dina Brooks, Elizabeth L. Inness, William E. McIlroy, George Mochizuki

**Affiliations:** Toronto Rehabilitation Institute, University Health Network, Room 11-117, 550 University Avenue, Toronto, ON M5G 2A2 Canada; Heart and Stroke Foundation Canadian Partnership for Stroke Recovery, Ottawa, ON Canada; Department of Physical Therapy and Rehabilitation Sciences Institute, University of Toronto, Toronto, ON Canada; Brain Sciences Program, Sunnybrook Research Institute, Toronto, ON Canada; Department of Kinesiology, University of Waterloo, Waterloo, ON Canada; St Joseph’s Healthcare Hamilton, Hamilton, ON Canada

**Keywords:** Stroke, Rehabilitation, Accidental falls, Postural balance

## Abstract

**Background:**

Falls are one of the most common medical complications post-stroke. Physical exercise, particularly exercise that challenges balance, reduces the risk of falls among healthy and frail older adults. However, exercise has not proven effective for preventing falls post-stroke. Falls ultimately occur when an individual fails to recover from a loss of balance. Thus, training to specifically improve reactive balance control could prevent falls. Perturbation training aims to improve reactive balance control by repeatedly exposing participants to postural perturbations. There is emerging evidence that perturbation training reduces fall rates among individuals with neurological conditions, such as Parkinson disease. The primary aim of this work is to determine if perturbation-based balance training can reduce occurrence of falls in daily life among individuals with chronic stroke. Secondary objectives are to determine the effect of perturbation training on balance confidence and activity restriction, and functional balance and mobility.

**Methods/design:**

Individuals with chronic stroke will be recruited. Participants will be randomly assigned to one of two groups: 1) perturbation training, or 2) ‘traditional’ balance training. Perturbation training will involve both manual perturbations (e.g., a push or pull from a physiotherapist), and rapid voluntary movements to cause a loss of balance. Training will occur twice per week for 6 weeks. Participants will record falls and activity for 12 months following completion of the training program. Standardized clinical tools will be used to assess functional balance and mobility, and balance confidence before and after training.

**Discussion:**

Falls are a significant problem for those with stroke. Despite the large body of work demonstrating effective interventions, such as exercise, for preventing falls in other populations, there is little evidence for interventions that prevent falls post-stroke. The proposed study will investigate a novel and promising intervention: perturbation training. If effective, this training has the potential to not only prevent falls, but to also improve safe independent mobility and engagement in daily activities for those with stroke.

**Trial registration:**

Current Controlled Trials: ISRCTN05434601.

## Background

Stroke is the leading cause of adult disability, and frequent falling is one of the most common medical complications post-stroke [1–3], representing significant healthcare costs. Remaining active after stroke is essential to recovery, maintaining quality of life, and reducing secondary stroke risk [4]. However, impaired postural control is associated with a high incidence of falls, reduced willingness to walk independently, and reduced overall activity [5, 6]. Balance control challenges not only elevate falls risk, but also lead to fear of falling [7] and are important determinants in reduced overall activity and community integration [8].

Previous research shows the essential role for reactive balance control in maintaining balance and mobility [9]. While falls can be initiated by external factors (e.g. slippery floor or nudge) [10], the capacity to recapture balance and prevent falling is fundamentally determined by the effectiveness of balance reactions. Fixed support reactions maintain balance without a change in the base of support (e.g. ankle and hip movements) and can be useful for defending against small postural perturbations [9, 11]. However, it is change-in-support reactions, which involve rapid stepping and grasping movements, that are ultimately essential to prevent falling [12]. Records of naturally-occurring falls and near falls reveal the importance of reactive stepping [13, 14], and that this is not a reaction reserved solely for the most potent postural perturbations [12, 15].

Reactive stepping is characterized by: 1) extremely rapid onset and movement speed [16, 17]; 2) amplitude and trajectory scaled to the degree of instability [12]; and 3) ability to accommodate environmental circumstances [18, 19]. These characteristics put tremendous demands on those with stroke, making the control of such reactions difficult [20–25]. Impaired limb control that delays execution of compensatory steps, or dyscoordination that makes foot placement or weight bearing difficult, leads to increased falls risk [24, 26, 27] and elevated fear and anxiety.

Despite control challenges associated with executing reactive steps, individuals with impaired balance control are increasingly dependent on these stepping responses because they are the last option to prevent falling. Because stroke survivors are at a high risk of falls, it is essential to develop approaches to re-train compensatory stepping after stroke. There is compelling evidence that physical exercise prevents falls among older adults [28]; however, no exercise intervention has effectively reduced risk of falls among individuals with stroke [29, 30]. More specific exercise, that is, perturbation training involving repeated exposure to applied balance disturbances, is necessary to achieve improvements in the control of fast reactive movements [22, 31–34]. Perturbation training is an emerging and promising treatment strategy for preventing falls [35]. The novelty of perturbation training is in the focus on speed of processing and execution of limb movements, as well as rapid restabilization; this differs from ‘traditional’ balance training programs using voluntary movements that allow participants to control speed. Such training is specific to the demands of balance recovery reactions to prevent a fall following a ‘real life’ loss of balance.

The primary objective of this study is to determine if a novel perturbation-based training program focused on improving reactive stepping in individuals with chronic stroke will reduce the risk for falls in the community. Secondary objectives are to determine the effect of perturbation training on balance confidence and activity restriction, and functional balance and mobility. Our primary hypothesis is that individuals with chronic stroke who complete perturbation training will be less likely to experience a fall up to 12-months following completion of the program, compared to individuals who complete a ‘traditional’ balance training program (control group). Our secondary hypothesis is that, compared to individuals in the control group, individuals with stroke who complete the perturbation training program will show: increased balance confidence; increased participation in daily activities in the year following completion of the program; and greater gains in functional balance and mobility.

## Methods

### Trial design

**Fig. 1:**
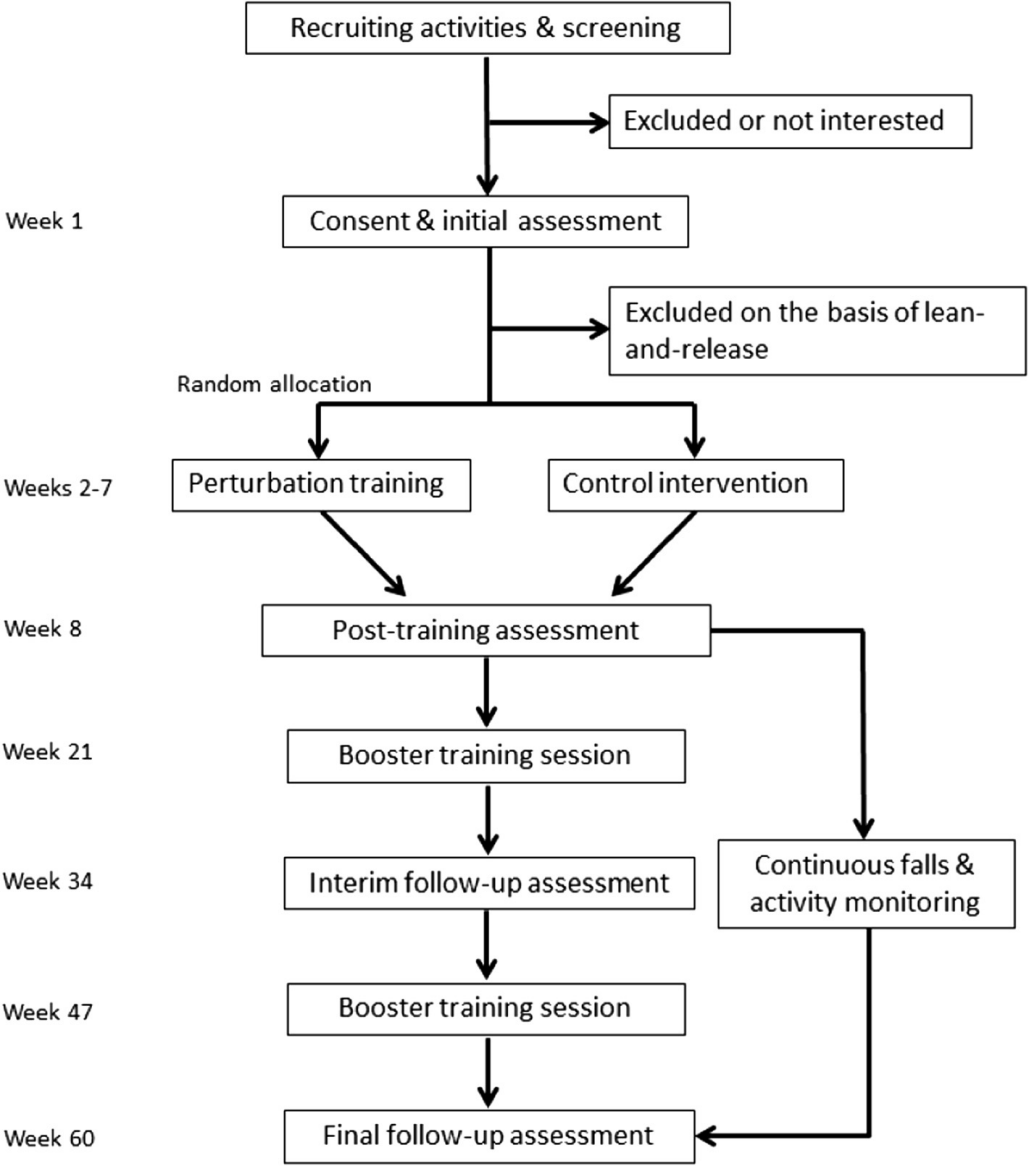
Study design flowchart. Following initial screening and consent, participants will undergo an initial assessment to confirm eligibility and facilitate group allocation. Eligible participants will be randomly assigned to either perturbation training or the control group. Immediately following completion of the training period, participants will repeat assessment of functional balance and mobility and balance confidence. Participants will then complete 12 months of regular falls and activity reporting. Participants will receive ‘booster’ training sessions 3 and 9 months after the initial training period. An interim follow-up assessment and final follow-up assessment will occur 6 and 12 months following the initial training period

This is a multi-site single-blind randomized controlled trial. Research activities will take place in two centres: 1) Toronto Rehabilitation Institute – University Centre site; and 2) Sunnybrook Health Sciences Centre. Individuals with chronic stroke will be recruited and randomly assigned to one of two groups: 1) perturbation training or 2) ‘traditional’ balance training (control). Participants will record falls, activity, and participation for 12 months following completion of the training program. Functional balance and mobility, and balance confidence will be assessed before and immediately after training, 6-months after the initial training, and at the end of the 12-month follow-up (Fig. 1).

### Participants

Community-dwelling individuals with chronic stroke (>6 months post-stroke) who are ≥50 years will be recruited. All participants will be able to stand independently without upper-limb support for >30 s and able to tolerate at least 10 postural perturbations while wearing a safety harness.

The following exclusion criteria will be applied: >2.1 m tall and/or weighing >150 kg (limits of the safety harness system); other neurological conditions that could affect balance control (e.g. Parkinson’s disease); lower extremity amputation; cognitive, language, or communication impairments affecting understanding instructions; recent (last 6 months) significant illness, injury or surgery; severe osteoporosis, defined by diagnosis of osteoporosis with fracture; poorly controlled diabetes or hypertension; contraindications to physical exercise, as identified using the Physical Activity Readiness Questionnaire [36]; currently attending in- or out-patient physiotherapy or other exercise targeting balance and mobility; and/or received perturbation training during formal rehabilitation in the 1 year prior to enrolment.

Eligible participants who have enrolled in research volunteer databases at participating sites will be invited to participate. Additionally, advertisements will be placed in the community (e.g., posters placed in participating sites, magazine advertisements, online advertisements) requesting volunteers for the study. Interested individuals will initially complete a telephone screening to determine eligibility. If the volunteer meets the criteria for the study, s/he will be scheduled to come to the site for an initial assessment. Prior to the initial assessment, the site research assistant will explain the study procedures again and will obtain written informed consent to participate.

**Fig. 2:**
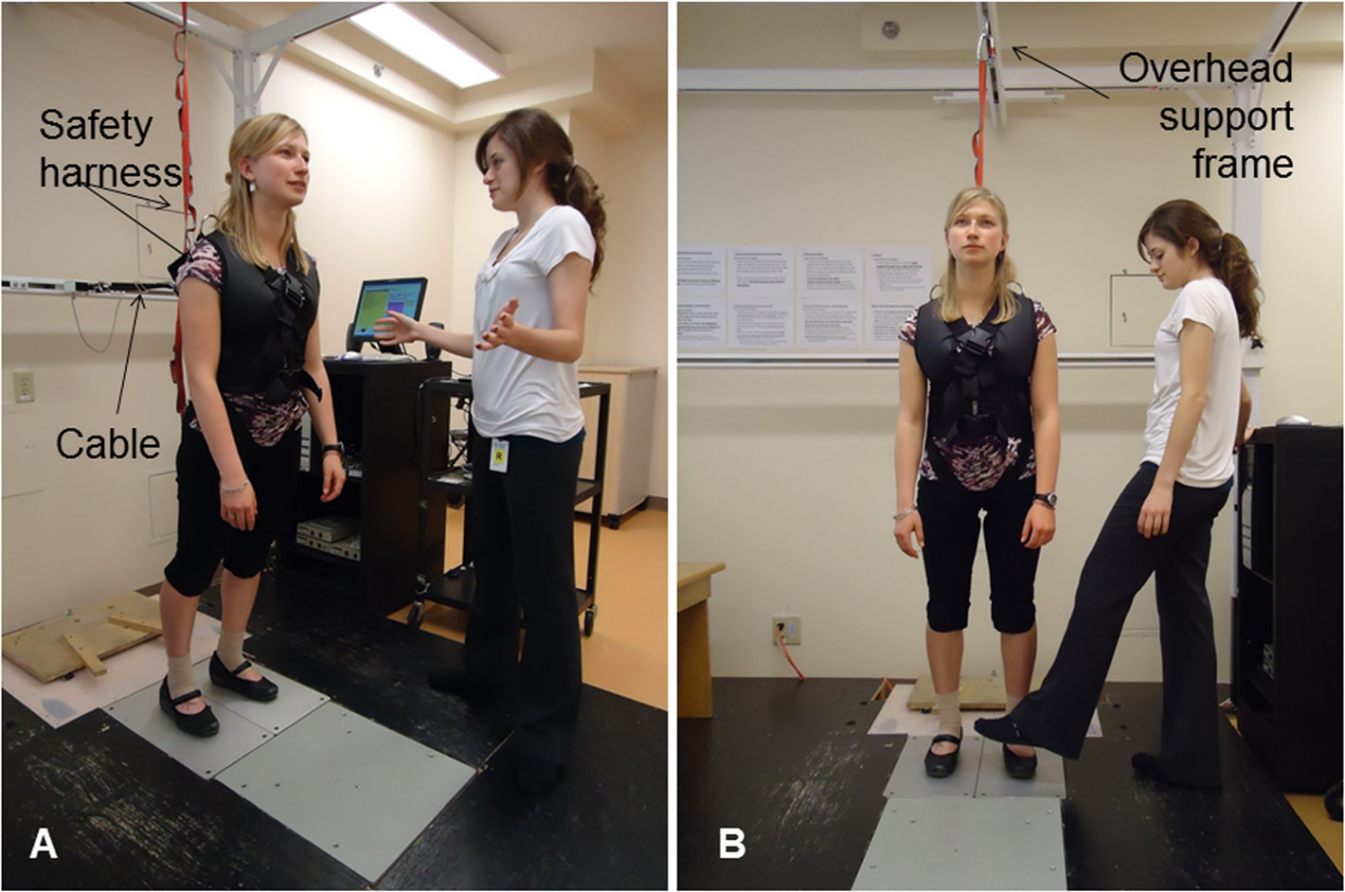
Lean-and-release postural perturbation. The participant leans forward so that approximately 10 % of body weight is supported by a cable attached to her back. At an unexpected time, the cable is released, causing the participant to start falling forward; a reactive step is required to regain stability. The research assistant stands close to provide assistance if the participant is unable to regain stability by stepping. A safety harness attached to an overhead support frame is worn, which prevents a fall to the floor. Panel **a** shows the ‘usual response’ condition where the participant is free to respond naturally. Panel **b** shows the ‘encouraged use’ condition; the preferred stepping limb (typically the non-paretic limb) is blocked, preventing step initiation with that limb and forcing use of the opposite limb to regain stability. (The individual shown is not a research participant. Consent was obtained for publication of the picture in this manuscript)

To confirm eligibility, participants will initially complete an assessment of reactive balance control using a lean-and-release postural perturbation of stance (Fig. 2; [37]). Two conditions will be completed: usual response, and encouraged use (5 trials in each condition). Participants will wear a harness attached to an overhead support and research staff will stand close to them to assist in the event of a failure to recover balance. Trials will be video-recorded for observational analysis.

As this is a longitudinal study, participants may be lost to follow-up. To help keep track of participants, we will request contact information of a friend or family member (‘alternative contact’). The alternative contact will only be contacted to obtain information about the whereabouts of a research participant if we are unable to contact him/her after multiple attempts.

### Group allocation and blinding

Participants will be assigned using blocked stratified randomization with allocation concealment to one of two training groups: 1) perturbation training, or 2) ‘traditional’ balance training (control). To maintain allocation concealment, a variable block size ranging from 4–8 will be used. There will be four strata based on two stratification factors: site (two levels), and frequency of ‘failures’ during baseline reactive balance control assessment (two levels). Stratification by site will ensure that the treatment groups are balanced within each institution accounting for potential differences in intervention administration between sites. Balance recovery ‘failures’ are defined as use of the overhead support harness and/or research assistant to prevent a fall to the floor, or stepping with the blocked limb during the encouraged-use condition; high frequency of failure predicts falls in the community [27]. Frequency of failures will be classified as either ‘high’ (≥10 % of trials) or ‘low’ (<10 % of trials). This approach will help to ensure that the groups do not differ significantly on this prognostic factor [38, 39], particularly within each site, and that there will be approximately equal numbers of participants assigned to each group. Group allocation will be performed centrally by the principal investigator, who will not be involved in recruiting, assessments, or administering the interventions. The random allocation sequence will be computer generated and maintained in an electronic file by the principal investigator.

Each site will be staffed by two individuals: a research assistant and a physiotherapist. The research assistant will be responsible for the initial telephone screening, recruiting, and performing pre- and post-intervention assessments; this individual will be blinded to group allocation. The physiotherapist will assist with assessments and administer the interventions. Participants will initially complete a test session to confirm eligibility for the study and to obtain baseline measures, including an assessment of reactive balance control. Upon completion of the initial assessment, information required for group allocation and information pertaining to participant eligibility will be communicated by the research assistant to the principal investigator. Upon randomization, the principal investigator will communicate group allocation to the physiotherapist. At the post-training, interim follow-up, and final follow-up assessments, the research assistant will be asked to guess the group allocation for each participant. The research assistant will also be asked to rate how confident they are in their guess of group allocation, and if they have received any information from the participant or other research personnel to violate blinding. If the research assistants’ guesses of group allocation are significantly greater than 50 % correct another researcher not involved with data collection or training will review the video-taped assessments and re-score the tests.

### Trial interventions

Interventions will be administered on a 1:1 basis (i.e., one physiotherapist per participant) by a trained and licensed physiotherapist. Interventions will follow a general guide but will be tailored to the individual participants’ ability, and individualized instructions and task modifications will be used to target participant-specific impairments in balance control. Participants will complete two 1 h training sessions per week for 6 weeks. Additionally, participants will be asked to return for two 1 h ‘booster’ training sessions 3 and 9 months following the initial training period. These booster sessions may help participants to retain some of the benefits of training [40, 41]. The proposed amount of training is similar to previous studies of perturbation training [32, 34, 42, 43]. To help to alleviate barriers to attending the program we will compensate participants for their travel expenses.

#### Group 1: ‘traditional’ balance training (control group)

The control group will complete a ‘traditional’ balance training program that focuses on maintaining stability during voluntary movement, rather than responding to instability. As previous research found no effect of such ‘traditional’ balance training on fall rates post-stroke [29, 30], we expect that the control participants will not be at a reduced risk of falling as a result of completing this program. Participants assigned to the control group will complete the Keep Moving with Stroke program [44]. This is an exercise program for individuals living in the community following stroke developed by a group of physiotherapists, and is based on balance and mobility interventions evaluated in clinical trials [45–48]. While the Keep Moving with Stroke program was initially designed to be delivered in a group format, it will be delivered 1:1 in the current study to match the attention received by the physiotherapist in the perturbation training program. Each session includes a 5−10 min warm-up followed by 40 min of mobility and balance-related exercises, and a 5−10 min cool-down with stretching. Full details of this program can be found online [44].

#### Group 2: perturbation training

The perturbation training program is grounded in motor learning principles of practice variability, practice order, feedback, guidance, instruction, and focus of attention, and in exercise physiology principles of overload, adaptation, progression, individualization, and specificity [31]. The initial assessment of reactive balance control will be used to identify participant-specific impairments in the control of reactive stepping; these impairments will then be targeted in the training program. A variety of tasks will be included to induce external or internal postural perturbations. External perturbations will be caused by forces outside the participants’ control (e.g., a push or pull from the physiotherapist). Internal perturbations are caused when the participant fails to control the centre of mass-base of support relationship during voluntary movement; ‘agility’ tasks, such as kicking a soccer ball, can be used to induce internal perturbations. Each session will include a 5−10 min warm-up, voluntary tasks that may induce internal perturbations, voluntary tasks combined with up to 60 external perturbations, and a 5−10 min cool-down. The number of repetitions is similar to previous studies [31, 32, 42], and our pilot testing indicates that individuals with sub-acute stroke can tolerate up to 30 perturbations in a 20 min session [22]. The task difficulty will be set such that participants will require an upper extremity response or external assistance (i.e., from the overhead harness or physiotherapist) or take more than 2 steps to regain stability approximately 50 % of the time. The progression in voluntary tasks occurs on a continuum from stable to mobile, and from predictable to unpredictable [49]. Additionally, progression occurs by increasing the magnitude of the external perturbation, or imposing sensory or environmental challenges. Examples of some of the training tasks are included in Table 1; a detailed training manual is available from the authors.

**Table 1:** Examples of voluntary tasks used in the perturbation training program

Week	Task type	Definition	Examples
1	Stable	Participants do not move their feet.	• Standing with eyes closed.
			• Shifting weight left/right or forward/back.
2-3	Quasi-mobile	Participants move their feet but remain ‘on the spot’.	• Rapid stepping forward and back.
			• ‘Walking’ in place.
4−5	Mobile	Participants move around the room.	• Walking forward or backward.
			• Side stepping.
6	Mobile & unpredictable	Participants move around the room in ways they cannot predict.	• Kicking a soccer ball against a wall.
			• Walking with sudden stops and changes in direction.

Tasks are completed alone and with external postural perturbations. External perturbation methods include ‘lean and release’ perturbations where participants lean forward, backward, left or right on the physiotherapists hands and are released suddenly (during stable tasks); a push or pull from the physiotherapist; or a trip with the physiotherapists foot (during mobile tasks). Each task can be modified to increase or reduce the difficulty, depending on participants’ abilities

#### Safety during training sessions

The physiotherapist will document activities in each session and deviations from recommended activities (e.g., due to participant fatigue). Participants will be asked to rate their perceived level of challenge (using a 5-point scale) at 10 min intervals throughout the sessions.

Blood pressure and heart rate will be measured and documented prior to the start of each session, and may be re-measured periodically throughout the session at the discretion of the physiotherapist. The session may be terminated if resting blood pressure or heart rate is outside of an acceptable range (systolic blood pressure: 90−140 mmHg; diastolic blood pressure: 60−90 mmHg; heart rate: 60−100 beats per minute). The decision to continue or terminate the study visit will be made by the physiotherapist, considering factors such as the participants’ usual resting blood pressure and heart rate, how far the measured values are outside of the acceptable range, the participants’ usual medications (e.g., beta-blockers), and the participants’ perception of how they are feeling. If the visit is terminated, the physiotherapist may advise that the participant follow-up with his/her primary care physician or may consult with on-site physicians.

Participants will be asked to bring their usual orthosis/brace, mobility aids, eyeglasses, and any medications taken on a *pro re nata* basis to training sessions. Participants will be deemed to have poor foot or ankle sensation and/or motor control if they meet any of the following criteria: Chedoke-McMaster Stroke Assessment (CMSA [50]) foot stage 3 or lower; unable to detect light touch with a cotton ball on fewer than 4 out of 5 trials; or unable to identify that that ankle has been placed into dorsiflexion, plantarflexion, inversion or eversion by the examiner on fewer than 8 out of 10 trials. Participants assigned to both groups with poor foot or ankle sensation and/or motor control who do not usually wear an ankle-foot orthosis will be required to wear an Aircast ankle brace for the training sessions to prevent ankle injury.

### Outcome measures

Participants will be followed for 12 months following completion of the initial training period. During this time, falls, activity, and participation will be reported regularly. Balance confidence and functional balance and mobility will be assessed at study enrolment, immediately after the initial training period, six-months after the initial training period, and at the end of the 12 months follow-up. All data will be collected by a trained research assistant who is blinded to group allocation. Table 2 summarizes that measures that will be taken at each time point.

**Table 2:** Summary of outcome measures and assessment time points

	Pre-training assessment	Post-training, interim and follow-up assessments	12-months follow-up period
Demographic and stroke information	✓		
Medical conditions	✓	✓	
Medications	✓	✓	
NIH-SS	✓		
CMSA	✓		
Lean-and-release test	✓		
BBS	✓	✓	
Mini-BES	✓	✓	
TUG	✓	✓	
ABC	✓	✓	
Falls reporting^a^	✓		✓
PASIPD^b^	✓		✓
SIPSO^b^	✓		✓

^a^Reported continuously throughout the 12-months follow-up period

^b^Questionnaires completed approximately every 2-months throughout the 12-months follow-up period

#### Cohort descriptors

The following demographic and medical information will be recorded at the time of study enrolment in order to characterize the study cohort: age, sex, height, weight, time since stroke, lesion location, other medical conditions, prescription medications, the National Institutes of Health Stroke Scale (NIH-SS [51]), and CMSA foot and leg scores [50]. Information regarding past medical history will be obtained by self-report and, when possible, will be verified from the participants’ hospital charts. The NIH-SS is an 11-item scale that provides a gross measure of the effects and severity of stroke. The NIH-SS has shown good intra- (ICCs = 0.93) and inter-rater (ICCs = 0.95) reliability [52]. The CMSA assigns a score according to the level of motor recovery in the foot and leg, and is frequently used to evaluate motor impairment post-stroke in clinical settings. The CMSA foot and leg scores have good intra- (ICCs = 0.94-0.98) and inter-rater (ICCs = 0.85-0.96) reliability [50].

#### Primary outcome – falls

A fall is defined as “an event that results in a person coming to rest unintentionally on the ground or other lower level” [14]. Participants will complete a 12-month falls monitoring period after the initial 6-week training period. Participants will be provided stamped addressed postcards containing a calendar to record falls, which they will complete daily. Participants will be asked to return each postcard to the research team fortnightly. Participants will receive a monthly study newsletter by mail containing health-related articles of interest, as well as a reminder to complete the postcards. If a participant does not return a postcard within two weeks, the research assistant will call them. In this telephone call, the research assistant will try to ascertain if the participant has experienced a fall in the previous two weeks. This method is considered the ‘gold standard’ for falls reporting [53]. Participants who report a fall on the calendar will be contacted by the research assistant to complete a short questionnaire in order to determine the cause and consequences of the fall.

#### Secondary outcomes (follow-up) – activity and participation

Fall rates may increase with increasing physical activity and mobility [54–60]. It is likely that when an individual with reduced balance control attempts to mobilize, s/he is at increased risk of losing balance and falling [61, 62]. Therefore, it is important to determine if fall rates are influenced by physical activity [63]. Conversely, improved balance control and reduced fear of falling should increase participation in activities. Physical activity and participation will be evaluated with the Physical Activity Scale for Individuals with Physical Disabilities (PASIPD [64]) and the Subjective Index of Physical and Social Outcome (SIPSO [65]), respectively, at six time points (every two months) during the 12-month follow-up. Regular administration of the questionnaires will provide an estimate of physical activity and participation over the duration of the follow-up period. The PASIPD is a 13-item questionnaire in which participants are asked to indicate the frequency and duration of recreational, household, and occupational physical activities completed in the previous 7 days. The PASID has been validated within a group of individuals with various physical disabilities, including those with stroke, showing good test-retest reliability (ρ = 0.77) and criterion validity when compared to accelerometer-based activity monitoring (ρ = 0.30; [66]). The SIPSO is a 10-item questionnaire that evaluates physical and social integration and participation in ‘normal’ daily life [65]. The SIPSO has good internal consistency (Cronbach’s α = 0.92) and test-retest reliability (ICC = 0.91) among those with stroke [65].

#### Secondary outcomes (pre-post) – functional balance and mobility, and balance confidence

Pre-post measures will be obtained immediately before and after the initial period of training, and 6 and 12 months after the end of the initial training period. Functional balance and mobility will be assessed using the Berg balance scale (BBS [67]), the mini-Balance Evaluation Systems test (mini-BES [68]), and the ‘Timed Up & Go’ (TUG [69]). The BBS is a 14-item observational rating scale that provides a measure of functional balance. Participants will be asked to perform each of the 14 tasks, and their ability to perform each task will be rated on a scale from 0–4. The BBS shows good internal consistency (Cronbach’s α = 0.92–0.98) and good inter-rater (ICCs = 0.95–0.98), intra-rater (ICCs = 0.97), and test-retest (ICC = 0.98) reliability in the stroke population [70]. The mini-BES is a 14-item observational rating scale that assesses systems underlying balance control, including reactive balance control and dynamic stability during walking [68]. The mini-BES has good inter- (ICC = 0.96) and intra-rater reliability (ICC = 0.97) among individuals with stroke [71]. The BBS is commonly used in balance training studies and clinical settings; therefore, inclusion of this measure will allow for comparison between the proposed and previous trials. The mini-BES is a more specific measure and is less susceptible to floor and ceiling effects than the BBS [71]; thus, the mini-BES may be more likely to reveal training-related changes in reactive control and dynamic stability during walking. The TUG is a frequently-used test of functional mobility that is related to falls risk [72–74]. Participants will be seated in a 45 cm high chair with armrests, and will be instructed to rise from the chair, walk “as quickly as is safe” to a marker placed 3 m directly in front of the chair, to circle the marker, and then to return to the chair. Participants will be allowed to use the armrests to assist them with rising from the chair only if absolutely necessary. The time taken from the start command until the participant sits back in the chair will be measured. The TUG has excellent test-retest reliability among individuals with stroke (ICC = 0.95; [75]). The Activity-specific Balance Confidence (ABC) questionnaire [76] will be used to assess balance confidence during daily activities before and after training. The ABC asks participants to rate, on a scale from 0–100 %, how confident they would be performing 16 everyday tasks. The ABC shows good internal consistency (Cronbach’s α = 0.94) and test-retest reliability (ICC = 0.85) in individuals with stroke [77].

### Statistical analysis and sample size

Negative binomial regression will be used to compare fall rates between the two groups and logistic regression will be used to compare the proportion of fallers between the two groups. If the groups differ on prognostic factors (e.g., stroke severity) or on physical activity during the follow-up period (i.e., PASIPD scores) then multiple negative binomial/logistic regression will be used. Intent-to-treat analysis will be used; all individuals with some falls-monitoring data will be included in the analysis. Survival analysis may be considered if a large number of individuals do not complete the entire 12-month follow-up. Exploratory per-protocol analysis and/or comparison of fall rates between those who return for the ‘booster’ training sessions and those who do not may also be conducted. Repeated-measures analysis of variance, with group-by-time interaction, will be used to evaluate the effect of the interventions on secondary outcome measures (e.g., functional balance measures, balance confidence). The interaction effect will reveal if there is a greater pre- to post-training improvement in the perturbation training group compared to the control group.

The target sample size has been estimated using a formula for negative binomial regression described elsewhere [78]. Assuming a rate of falls in the control group of 1.75 per person per year [27], a reduction in rate of falling of 46 % in the perturbation training group [35], mean follow-up time of 11 months per person (i.e., some participants will be lost to follow-up before 12 months), level of significance (α) of 0.05, and power (1-β) of 0.8, we estimate that 37 participants per group will be required to show significant effects. From previous studies [32, 79], we expect that ~20 % of individuals will withdraw prior to completing the intervention. While many of these individuals will be willing to complete the 12-month falls monitoring period and could be included in intent-to-treat analysis, we conservatively assume that 20 % of individuals will withdraw from the study. Therefore, we will aim to recruit at least 46 participants per group (i.e., 92 participants total).

### Safety monitoring

A Safety and Monitoring Committee will be established to ensure that patient safety is maintained by monitoring the trial for possible harmful effects of the intervention (e.g., falls and injuries associated with the intervention). The committee will evaluate the data on adverse events in order to recommend whether the study should continue, be modified, or stopped for safety concerns. The Safety and Monitoring Committee will be an independent multidisciplinary group of 4 members with research and/or clinical experience in rehabilitation post-stroke. Membership will last until the trial is complete. The members will be free of major financial or intellectual conflicts of interest that could prevent them from objectively reviewing the interim data and giving advice. The Safety and Monitoring Committee will meet once prior to the initiation of the trial to discuss and agree on the mandate, and then twice per year until the end of the study to evaluate accrued data on adverse events. Additional meetings may be held if deemed necessary by members of the committee.

## Discussion

Despite the high fall rates experienced by individuals with stroke, and the corresponding increased fear of falling and reduced mobility, there is currently a paucity of research determining how to prevent falls and still maintain independent mobility in this population. While several studies demonstrate the efficacy of exercise for preventing falls in older adults, no study has found that exercise prevents falls among individuals with stroke. It is our view that the capacity for the control of rapid balance reactions is the primary factor that contributes to elevated fall risk and poor balance confidence post-stroke. However, little work has been done to understand the effects of training on reactive balance control. We believe that this novel training paradigm focus of study has the potential to contribute significantly to current knowledge regarding physiotherapy best practices for prevention of falls post-stroke.

## Abbreviations

ABC: activity-specific balance confidence scale
BBS: Berg balance scale
Mini-BES: Mini-balance evaluation systems test
NIH-SS: National Institutes of Health stroke scale
PASIPD: physical activity scale for individuals with physical disabilities
SIPSO: subjective index of physical and social outcome
TUG: timed-up and go
